# RETRACTION: Identification of Recurrence‐Related MicroRNAs in Hepatocellular Carcinoma Following Liver Transplantation

**DOI:** 10.1002/1878-0261.70313

**Published:** 2026-08-11

## Abstract

**RETRACTION:** Z.‐B. Han, L. Zhong, M.‐J. Teng, J.‐W. Fan, H.‐M. Tang, J.‐Y. Wu, H.‐Y. Chen, Z.‐W. Wang, G.‐Q. Qiu, and Z.‐H. Peng, “Identification of Recurrence‐Related MicroRNAs in Hepatocellular Carcinoma following Liver Transplantation,” *Molecular Oncology* 6, no. 4 (2012): 445‐457, https://doi.org/10.1016/j.molonc.2012.04.001.

This article, published online on 17 April 2012 in Wiley Online Library (wileyonlinelibrary.com), has been retracted by agreement between the journal Editor in Chief, Kevin Ryan; the Federation of European Biochemical Societies; and John Wiley and Sons Ltd.

A third‐party organization contacted the publisher regarding concerns pertaining to deceased donor organ sourcing practices in multiple articles, including this one. Credible reporting has been published widely, which indicates that research featuring organ transplantation in China did not comply with international ethics standards. As reported by Rogers et al. and others [1–3], concerns have been raised about the sourcing of organs for transplantation in China during the period relevant to this study, with questions about compliance with internationally accepted ethical standards and transparency in donor procurement practices at the time of publication.

The Materials and Methods section for this article indicate that the study reviewed data from liver transplantation patients between January 2002 and December 2007. The article states that the study protocols were approved by the Institutional Review Boards of Shanghai First People's Hospital and Shandong Provincial Qianfoshan Hospital and that informed consent was obtained from patients. No information regarding organ donor sources was provided. Because the entirety of liver transplant cases reported in this article occurred before the existence of a voluntary organ donation program, serious doubts remain regarding the origins of these donations. The authors did not respond to inquiries made by the publisher about these concerns.

Furthermore, a third party had reported on PubPeer [4] that the miR‐24 and miR‐126 graphs in Figure 2A were duplicated despite showing different experimental conditions. Further investigation by the journal editors found that the miR0886‐5p and miR‐147 graphs in Figure 5A and the miR‐147 and miR‐223 graphs in Figure 2B contained additional data overlap. Unexpected similarities were also found in portions of the graphs between Figures 3A and 3B and between Figures 4A and 4B.

The publisher and editors cannot verify the ethics committee registration or the informed consent protocols for the study reported in this article. Furthermore, questions remain as to whether the organ donations reported in the article complied with internationally recognized ethical guidelines in place at the time of publication. Lastly, evidence of data duplication and manipulation compromises the editors' confidence in the results presented in this article. Therefore, the journal has decided to issue a retraction for this article.

The authors were informed of this retraction.

References

[1] W. Rogers, M. P. Robertson, A. Ballantyne, B. Blakely, R. Catsanos, R. Clay‐Williams, and M. F. Singh, “Compliance with Ethical Standards in the Reporting of Donor Sources and Ethics Review in Peer‐Reviewed Publications Involving Organ Transplantation in China: a Scoping Review,” *BMJ Open* 9, no. 2 (2019): 10.1136/bmjopen-2018-024473.PMC637753230723071

[2] S. Dai and L. Xu, “China to stop using organs from executed prisoners for transplantations,” *BMJ* (2015): 350, 10.1136/bmj.h239.25596584

[3] J. Huang, H. Wang, S. T. Fan, B. Zhao, Z. Zhangm L. Hao, F. Huo, and Y. Liu, “The National Program for Deceased Organ Donation in China,” *Transplantation Journal* 96, no. 1 (2013): 5‐9, 10.1097/TP.0b013e3182985491.23743728

[4] Unregistered Submission, Comments on “Identification of recurrence‐related microRNAs in hepatocellular carcinoma following liver transplantation,” PubPeer, April 2017. https://pubpeer.com/publications/BC8142D1977BB8D691ED59ABBCC512.

**RETRACTION:** Z.‐B. Han, L. Zhong, M.‐J. Teng, J.‐W. Fan, H.‐M. Tang, J.‐Y. Wu, H.‐Y. Chen, Z.‐W. Wang, G.‐Q. Qiu, and Z.‐H. Peng, “Identification of Recurrence‐Related MicroRNAs in Hepatocellular Carcinoma following Liver Transplantation,” *Molecular Oncology* 6, no. 4 (2012): 445‐457, https://doi.org/10.1016/j.molonc.2012.04.001.

This article, published online on 17 April 2012 in Wiley Online Library (wileyonlinelibrary.com), has been retracted by agreement between the journal Editor in Chief, Kevin Ryan; the Federation of European Biochemical Societies; and John Wiley and Sons Ltd.

A third‐party organization contacted the publisher regarding concerns pertaining to deceased donor organ sourcing practices in multiple articles, including this one. Credible reporting has been published widely, which indicates that research featuring organ transplantation in China did not comply with international ethics standards. As reported by Rogers et al. and others [1–3], concerns have been raised about the sourcing of organs for transplantation in China during the period relevant to this study, with questions about compliance with internationally accepted ethical standards and transparency in donor procurement practices at the time of publication.

The Materials and Methods section for this article indicate that the study reviewed data from liver transplantation patients between January 2002 and December 2007. The article states that the study protocols were approved by the Institutional Review Boards of Shanghai First People's Hospital and Shandong Provincial Qianfoshan Hospital and that informed consent was obtained from patients. No information regarding organ donor sources was provided. Because the entirety of liver transplant cases reported in this article occurred before the existence of a voluntary organ donation program, serious doubts remain regarding the origins of these donations. The authors did not respond to inquiries made by the publisher about these concerns.

Furthermore, a third party had reported on PubPeer [4] that the miR‐24 and miR‐126 graphs in Figure 2A were duplicated despite showing different experimental conditions. Further investigation by the journal editors found that the miR0886‐5p and miR‐147 graphs in Figure 5A and the miR‐147 and miR‐223 graphs in Figure 2B contained additional data overlap. Unexpected similarities were also found in portions of the graphs between Figures 3A and 3B and between Figures 4A and 4B.

The publisher and editors cannot verify the ethics committee registration or the informed consent protocols for the study reported in this article. Furthermore, questions remain as to whether the organ donations reported in the article complied with internationally recognized ethical guidelines in place at the time of publication. Lastly, evidence of data duplication and manipulation compromises the editors' confidence in the results presented in this article. Therefore, the journal has decided to issue a retraction for this article.

The authors were informed of this retraction.

**References**

[1] W. Rogers, M. P. Robertson, A. Ballantyne, B. Blakely, R. Catsanos, R. Clay‐Williams, and M. F. Singh, “Compliance with Ethical Standards in the Reporting of Donor Sources and Ethics Review in Peer‐Reviewed Publications Involving Organ Transplantation in China: a Scoping Review,” *BMJ Open* 9, no. 2 (2019): 10.1136/bmjopen-2018-024473.PMC637753230723071

[2] S. Dai and L. Xu, “China to stop using organs from executed prisoners for transplantations,” *BMJ* (2015): 350, 10.1136/bmj.h239.25596584

[3] J. Huang, H. Wang, S. T. Fan, B. Zhao, Z. Zhangm L. Hao, F. Huo, and Y. Liu, “The National Program for Deceased Organ Donation in China,” *Transplantation Journal* 96, no. 1 (2013): 5‐9, 10.1097/TP.0b013e3182985491.23743728

[4] Unregistered Submission, Comments on “Identification of recurrence‐related microRNAs in hepatocellular carcinoma following liver transplantation,” PubPeer, April 2017. https://pubpeer.com/publications/BC8142D1977BB8D691ED59ABBCC512.

